# CT-based methods for assessment of metabolic dysfunction associated with fatty liver disease

**DOI:** 10.1186/s41747-023-00387-0

**Published:** 2023-11-21

**Authors:** Na Hu, Gang Yan, Maowen Tang, Yuhui Wu, Fasong Song, Xing Xia, Lawrence Wing-Chi Chan, Pinggui Lei

**Affiliations:** 1 Department of Radiology, The Affiliated Hospital of Guizhou Medical University, Guiyang, China; 2 Department of Nuclear Medicine, The Affiliated Hospital of Guizhou Medical University, Guiyang, China; 3 Department of Health Technology and Informatics, The Hong Kong Polytechnic University, Kowloon, Hong Kong SAR China

**Keywords:** Biomarkers, Fatty liver, Liver cirrhosis, Liver diseases, Tomography (x-ray computed)

## Abstract

Metabolic dysfunction-associated fatty liver disease (MAFLD), previously called metabolic nonalcoholic fatty liver disease, is the most prevalent chronic liver disease worldwide. The multi-factorial nature of MAFLD severity is delineated through an intricate composite analysis of the grade of activity in concert with the stage of fibrosis. Despite the preeminence of liver biopsy as the diagnostic and staging reference standard, its invasive nature, pronounced interobserver variability, and potential for deleterious effects (encompassing pain, infection, and even fatality) underscore the need for viable alternatives. We reviewed computed tomography (CT)-based methods for hepatic steatosis quantification (liver-to-spleen ratio; single-energy “quantitative” CT; dual-energy CT; deep learning-based methods; photon-counting CT) and hepatic fibrosis staging (morphology-based CT methods; contrast-enhanced CT biomarkers; dedicated postprocessing methods including liver surface nodularity, liver segmental volume ratio, texture analysis, deep learning methods, and radiomics). For dual-energy and photon-counting CT, the role of virtual non-contrast images and material decomposition is illustrated. For contrast-enhanced CT, normalized iodine concentration and extracellular volume fraction are explained. The applicability and salience of these approaches for clinical diagnosis and quantification of MAFLD are discussed.

**Relevance statement**

CT offers a variety of methods for the assessment of metabolic dysfunction-associated fatty liver disease by quantifying steatosis and staging fibrosis.

**Key points**

• MAFLD is the most prevalent chronic liver disease worldwide and is rapidly increasing.

• Both hardware and software CT advances with high potential for MAFLD assessment have been observed in the last two decades.

• Effective estimate of liver steatosis and staging of liver fibrosis can be possible through CT.

**Graphical Abstract:**

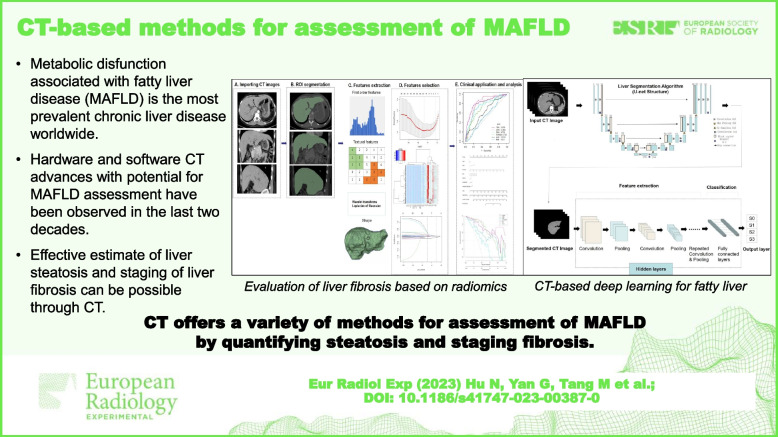

## Background

Metabolic dysfunction-associated fatty liver disease (MAFLD), previously called metabolic nonalcoholic fatty liver disease, NAFLD, is a major risk factor for chronic liver disease, which affects approximately a quarter of the population worldwide [1, 2]. It is characterized by a pathological spectrum of severity with steatosis exceeding 5% in the hepatocytes, including alcohol-induced steatosis or concomitant secondary hepatic fat accumulation [1]. MAFLD can range from simple hepatocellular steatosis to steatohepatitis and liver fibrosis, which ultimately may lead to hepatocellular carcinoma, liver failure, and even death [3]. Furthermore, MAFLD is strongly linked to the occurrence and development of cardiovascular diseases [4].

MAFLD is mainly pathologically characterized by hepatocyte steatosis, hepatocyte ballooning degeneration, lobular inflammation, and fibrosis. The severity of MAFLD is best described by combining the stage of fibrosis with the grade of activity [1]. The degree of liver fibrosis is a crucial independent prognostic factor for mortality and morbidity due to liver disease in MAFLD patients. Therefore, an accurate assessment of hepatic steatosis and fibrosis is crucial in the diagnosis and treatment of MAFLD [5]. Liver biopsy has long been the reference standard for accurately evaluating steatosis and the degree of fibrosis [6]. Nevertheless, liver biopsy has some limitations, including sampling error, intra- and inter-observer variability, and invasiveness, which is associated with risks such as infection, pain, perforation of the organs near the liver, bleeding and, in rare cases, even death [7]. As such, it is essential to develop practical, robust, and cost-effective tests for the diagnosis, staging, and monitoring of MAFLD. Non-invasive modalities based on serum markers and imaging examinations, which circumvent the limitations of liver biopsy, have been developed for routine use in clinical practice [8, 9].

**Fig. 1:**
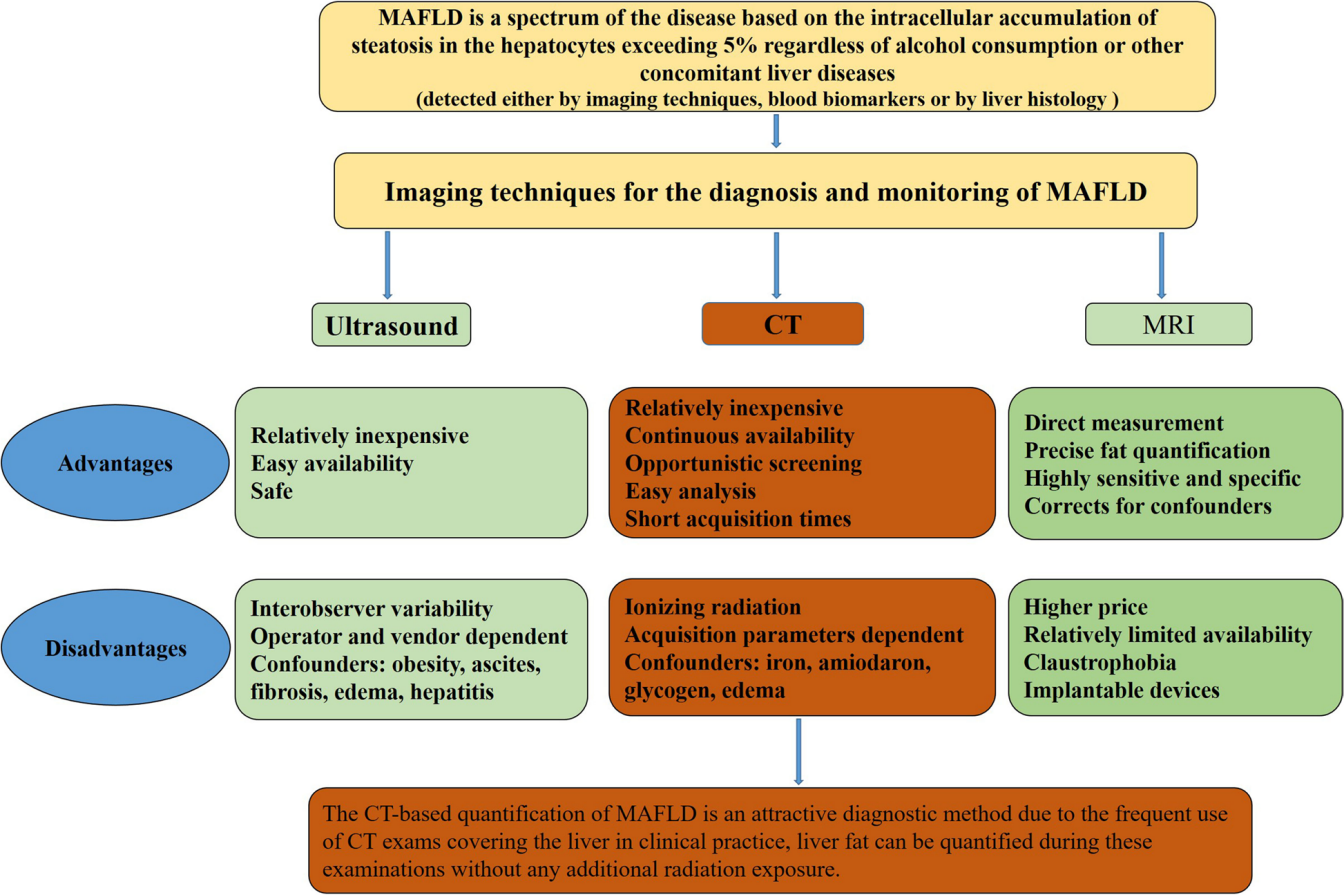
Comparison of ultrasound, CT, and MR for the diagnosis and monitoring of MAFLD. *MAFLD* Metabolic dysfunction-associated fatty liver disease, *CT* Computed tomography, *MRI* Magnetic resonance imaging, *US* Ultrasound

Imaging techniques have been used for the evaluation of steatosis and assessment of liver fibrosis severity in MAFLD for nearly two decades (Fig. 1). The current reference standards for non-invasive measurement of hepatic steatosis include magnetic resonance spectroscopy and magnetic resonance imaging-proton density fat fraction (MRI-PDFF) [10, 11]. However, their high cost and limited availability limit their widespread use in clinical practice. Ultrasound has been widely used to assess hepatic steatosis in clinical settings because of its low cost and availability. Emerging quantitative ultrasound elastographic techniques are also being developed and validated for the diagnosis of hepatic steatosis and fibrosis [12–14]. However, the accuracy of ultrasound-based methods is affected by various factors, such as the level of obesity and the serum alanine aminotransferase [15].

CT can also measure liver fat [16, 17] and has been proven to be effective for detecting steatosis [17–21], but it exposes patients to ionizing radiation. Nonetheless, the CT-based quantification of MAFLD is an attractive diagnostic method as CT exams including the liver are common in clinical practice and it can be performed to quantify liver fat without additional radiation exposure. Furthermore, CT-based imaging biomarkers are increasingly used to diagnose and stage hepatic fibrosis [22–26], as they can be retrieved quantitatively, retrospectively, and rapidly using automated systems [24, 25]. Hence, CT liver fat measurement could be an effective method for the screening and diagnosis of MAFLD. This article reviews recent studies on CT techniques for hepatic steatosis quantification and CT-based tools for staging hepatic fibrosis and discusses their practical application in routine clinical diagnosis and quantification of MAFLD.

### Estimation of liver steatosis

The traditional methods of conventional CT diagnosis of hepatic steatosis are based on liver Hounsfield units (HU) difference between liver and spleen, typically the liver-to-spleen ratio. These methods classify steatosis as normal, mild, moderate, or severe [19, 26–29]. HU is a unit of measurement used to measure the density of a local tissue or organ in the body as seen in CT scan. HU can be calculated using the following formula:

$$\mathrm{HU}=\left(\frac{\begin{array}{ccc}{\mu }_{\mathrm{material}}& -& {\mu }_{\mathrm{water}}\end{array}}{\begin{array}{ccc}{\mu }_{\mathrm{water}}& -& {\mu }_{\mathrm{air}}\end{array}}\right)\times 1000$$

where μ is the CT linear attenuation coefficient and μ_air_ is almost zero and can be ignored

However, these methods merely provide a qualitative or semi-quantitative assessment of liver fat content and have been deemed accurate for moderate-to-severe steatosis but insensitive to mild steatosis [21]. Additionally, the outcomes are susceptible to variations in scanning conditions, including different tube voltages and usage of CT scanners from various manufacturers [30]. Furthermore, these evaluation methods cannot detect early stages of liver fibrosis based on fatty liver. Recently, various quantitative and reliable technologies based on CT have been developed and validated for evaluating the presence of steatosis and have high accuracy than the traditional methods of conventional CT. We summarized the CT-based technologies for quantitative evaluation of hepatic steatosis in Table 1, along with several representative studies presented in Table 2.

**Table 1:** Quantitative evaluation of hepatic steatosis using computed tomography

CT-based tools	Principles	Acquisition methods
Deep learning	Automated algorithms for liver segmentation and analysis	All voxels designated as liver by the segmentation algorithm were analyzed, and the mean and median HU were computed
Quantitative CT	Using a scanner with a five-rod calibration phantom with an aqueous K_2_HPO_4_ bone density standard placed beneath the participants	CTFF = (HU_lean_ -HU_liuer_)/(HU_lean_—HU_fat_) HU_liver_ is the measurement in Hounsfield units in the liver HU_lean_ is the value in Hounsfield units for fat-free liver tissue HU_fat_ is the value for 100% fat
Dual-energy CT	It provides information about tissue composition	VNC and iodine maps; TNC images; MMD algorithm
Deep learning	Automated algorithms for liver segmentation and analysis	All voxels designated as liver by the segmentation algorithm were analyzed, and the mean and median HU were computed
Photon-counting CT	It is able to detect and weight individual photons based on their energies	TNC and VNC images

*CT* Computed tomography, *CTFF* CT fat fraction, *HU* Hounsfield units, *MMD* Multi-material decomposition, *TNC* True non-contrast, *VNC* Virtual non-contrast

**Table 2:** Summary of CT studies for quantitative evaluation of hepatic steatosis

First author [Reference]	Number of patients	Methods	Reference standard	AUROC or positive and negative predictive value	Sensitivity	Specificity
Pickhardt [31]	1,204	Deep learning	MR-PDFF	Steatosis ≥ 5%: 0.669 Steatosis ≥ 10%: 0.854 Steatosis ≥ 15%: 0.962	Steatosis ≥ 5%: 34.0% Steatosis ≥ 15%:75.9%	Steatosis ≥ 5%: 94.2% Steatosis ≥ 10%: 95.7%
Guo [16]	400	QCT	MR-PDFF	Steatosis ≥ 5%: 0.87 Steatosis ≥ 14%: 0.99	Steatosis ≥ 5%:75.9% Steatosis ≥ 14%:84.8%	Steatosis ≥ 5%: 83.3% Steatosis ≥ 14%: 98.4%
Hyodo [32]	33	DECT FVF	Histologic	FVF discrimination between histologic grade 0 and grades 1–3: 0.88	Cut-off 4.6% for FVF: 82%	Cut-off 4.6% for FVF: 100%
Cao [33]	50	DECT MMD	Pathological	FVF correlated well with the pathological grades: 0.92	89.2%	100%
Zhang [34]	128	DECT VNC	MR-PDFF	Steatosis > 6%: 0.834 and 0.872 in the right and left lobe	57%/93.9% (right)	67.9%/90% (left)
Niehoff [35]	140	PCD-CT VNC	Previous reported cut-off values for diagnosing hepatic steatosis (CT (L) ≤ 40 HU, CT (L-S) ≤ -10 HU, CT (L/S) ≤ 0.8	PPV and NPV for the detection of hepatic steatosis: 30% and 99.5% When adjusting cut-off values: 41% and 99.6%	PPV and NPV: 94% When adjusting cut-off values: 94%	PPV and NPV: 87% When adjusting cut-off values: 92%

*AUROC* Area under the receiver operating characteristic curve, *DECT* Dual-energy computed tomography, *FVF* Fat volume fraction, *HU* Hounsfield units, *MMD* Multi-material decomposition, *MR-PDFF* Magnetic resonance imaging-derived proton density fat fraction, *NPV* Negative predictive value, *PPV* Positive predictive values, *QCT* Quantitative CT, *VNC* Virtual non-contrast

### Single-energy “quantitative” CT (QCT)

This technique was initially developed to measure bone mineral density [36]. QCT converts HU measurements into tissue densities by scanning a phantom with standards corresponding to the known density of bone and soft tissue [37]. Using QCT phantom, which includes water and fat standards, CT HU can be used to estimate tissue fat content, as adiposity negatively correlated with decreasing HU. In a study, a 120-kVp QCT scan of the liver was used to measure the fat content. Single-energy QCT-derived percentage of liver fat content was calculated using the following equation [37]:

$$\mathrm{\%fat}=\left\{\frac{\begin{array}{ccc}{\mathrm{HU}}_{\mathrm{lean}}& -& {\mathrm{HU}}_{\mathrm{liver}}\end{array}}{\begin{array}{ccc}{\mathrm{HU}}_{\mathrm{lean}}& -& {\mathrm{HU}}_{\mathrm{fat}}\end{array}}\right\}\times 100\%$$

Compared with traditional semiquantitative CT approaches, QCT can directly measure liver fat content and the calibration phantom can be used for multi-center studies. QCT significantly decreases the variability in HU measurements due to factors such as x-ray filtration, kVp, patient size, and splenic HU variation.

Peripheral QCT has been used in small animal models to assess body and liver fat [38]. Xu et al. [39] verified this method by comparing QCT liver fat measurements in goose liver samples with those obtained from biochemical analysis and chemical shift-encoded MRI. Guo et al. [16] validated the accuracy of QCT in measuring hepatic steatosis content using chemical shift-encoded MRI-PDFF as a standard in a large prospective cohort of healthy individuals. Furthermore, in a subsequent study, the researchers compared the prevalence of hepatic steatosis among Chinese and American cohorts using QCT measurements and found a strong correlation between the QCT liver fat measurement and MRI-PDFF determined using the mDixon Quant software [40]. Those studies collectively demonstrated the potential of quantitative computed tomography (QCT) as a reliable and accurate method for hepatic steatosis quantification. The findings underscore its usefulness in noninvasively assessing liver fat content in various cohorts. QCT holds promise as a valuable tool in clinical and research settings for hepatic steatosis evaluation. Further investigations and standardized protocols will aid in its widespread adoption and integration into routine clinical practice.

### Dual-energy CT (DECT)

This is a qualitative and quantitative modality that obtains multi-material decomposition based on the attenuation measurements of x-rays at multiple diverse energies to differentiate and quantify the composition of the target [41]. Over the past decade, DECT has been increasingly employed for quantifying hepatic steatosis in phantom, animal, and clinical studies and showing promise over conventional CT imaging due to its ability to accurately quantify fat content [42–44].

However, Artz et al. [45] reported that the fat (water) content measurements strongly correlated with triglycerides in a phantom but not as well *in vivo*. Additionally, there have been differing opinions on the superiority of DECT over conventional single-energy CT and contrast-enhanced DECT for quantitatively assessing liver steatosis [18, 46]. Despite these variations, several principal studies have demonstrated the accuracy and reproducibility of DECT for quantitative assessment of liver fat, making it suitable for clinical use [32, 33, 47]. Zhang et al. [34] demonstrated that attenuation at virtual non-contrast (VNC) images of DECT had a moderate correlation with liver fat content and > 90% specificity for diagnosis attenuation at virtual non-contrast (VNC) images of DECT had a moderate correlation with liver fat content and > 90% specificity for diagnosis in fatty liver. In another research by Molwitz et al. [48] developed a fat quantification method based on dual-layer detector-based spectral, a detector-based DECT scanner, which demonstrated strong agreement with MRI techniques for patient liver and muscle.

By focusing on these principal studies, we can better understand the strengths and limitations of DECT in quantifying liver fat and appreciate its potential clinical utility. Contrast-enhanced DECT demonstrates high specificity in evaluating hepatic steatosis through VNC attenuation of the liver, making it a promising tool for the early and incidental detection of fatty liver disease. However, hepatic iron deposition might be the most significant influencing factor for DECT in the quantitative assessment of liver steatosis. The potential for future application of an iron-specific multi-material decomposition algorithm in DECT may enable quantitative assessment of liver steatosis while effectively correcting for the influences of iron and iodine in the liver.

### Deep learning (DL)-based methods

**Fig. 2:**
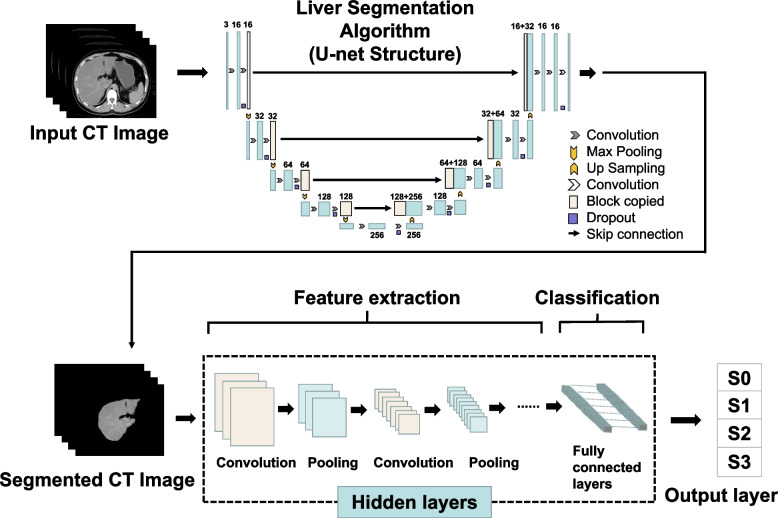
Flowchart of deep learning for fatty liver. *CT* Computed tomography

The application of artificial intelligence, in particular machine learning, has improved the accuracy of MAFLD diagnostic techniques. DL is a branch of machine learning commonly using convolutional neural networks. In Fig. 2, a flowchart of DL methods for liver assessment, which includes three layers is shown: input, hidden, and output. The input liver image is automatically delineated by the U-net structure. The hidden layers perform convolution and pooling of images, which are then fed to the fully connected layers. To generate high-dimensional manageable features, convolution and pooling of input images are repeated before feeding analyzed features of input imaged into fully connected layers for the classification task. Finally, probabilities for the classes are returned by the output layer. The loss function was calculated as follows:

$$\mathrm{Loss}=1-\frac{2{\sum }_{\mathrm{c}=1}^{\mathrm{C}}{\mathrm{w}}_{\mathrm{c}}{\sum }_{\mathrm{m}=1}^{\mathrm{M}}{\mathrm{P}}_{\mathrm{cm}}{\mathrm{G}}_{\mathrm{cm}}}{{\sum }_{\mathrm{c}=1}^{\mathrm{C}}{\mathrm{w}}_{\mathrm{c}}{\sum }_{\mathrm{m}=1}^{\mathrm{M}}\left({\mathrm{P}}_{\mathrm{cm}}^{2}+{\mathrm{G}}_{\mathrm{cm}}^{2}\right)}$$

$${\mathrm{w}}_{\mathrm{c}}=\frac{1}{{({\sum }_{\mathrm{m}=1}^{\mathrm{M}}{\mathrm{G}}_{\mathrm{cm}})}^{2}}$$

where $P$ represents the predicted image and $\mathrm{G}$ denotes the corresponding ground truth; $C$ represents the number of classes; $\mathrm{M}$ represents the number of elements in $P$ or $\mathrm{G}$’s first two dimensions; and ${\mathrm{w}}_{\mathrm{c}}$ represents the weighting factor for each class. The Dice coefficient is calculated using the following formula:

$$\mathrm{Dice}=2\frac{{\mathrm{P}}_{\mathrm{c}}\cap {\mathrm{G}}_{\mathrm{c}}}{{\mathrm{P}}_{\mathrm{c}}+{\mathrm{G}}_{\mathrm{c}} }$$

where ${P}_{\mathrm{c}}$ and ${G}_{\mathrm{c}}$ represent the predicted image and ground truth of each class, respectively ($C$ = 1, 2). For each class, Jaccard’s index is calculated as follows:

$$\mathrm{Jaccard}=\frac{{\mathrm{P}}_{\mathrm{c}}\cap {\mathrm{G}}_{\mathrm{c}}}{{\mathrm{P}}_{\mathrm{c}}\cup {\mathrm{G}}_{\mathrm{c}}}$$

Several studies have evaluated the performance of DL-based CT in liver fat quantification for MAFLD assessment in recent years. Kullberg et al. [49] used DL to analyze CT data to develop and validate an automated image-processing technique for analyzing body composition, including liver fat. Graffy et al. [25] proposed an automated liver segmentation tool based on deep learning was validated by retrospectively quantifying liver fat in 9,552 consecutive patients. In other studies, DL volumetric liver segmentation algorithm was used to evaluate liver fat based on contrast-enhanced CT images, which achieved high accuracy as an objective tool for assessing hepatic steatosis [31]. However, as this method does not exclude liver vessels, which have a higher HU value, it may overestimate liver attenuation. To reduce the vessel effects, Huo et al. [50] proposed a method that combines deep learning and morphological operations for accurate estimation the liver attenuation in peripheral regions of interest. Overall, these studies show the potential of deep learning technology for segmentation, quantification, and standardization of diagnosis in patients with MAFLD. In the future, this fully automated CT tool may be used in investigations with larger retrospective cohorts since it provides both rapid and objective assessment.

### Photon-counting CT (PCCT)

In 2021, the first clinical PCCT scanner using a photon-counting detector with quantum technology to enhance the capability of spectral imaging, was introduced, taking CT technology to the next level. This has enabled PCCT technology to be used for true multi-energy CT scanning, as demonstrated by several preclinical and clinical studies [51, 52]. PCCT is an evolution in CT data collection methods within the realm of energy. It can produce material-specific or virtual monoenergetic images from CT data similar to DECT. Compared with conventional CT detectors, photon-counting detectors can detect and measure single photons and their energy because they are composed of one thick layer of semiconductor material [53, 54]. In addition, in contrast to DECT, PCCT has the potential to improve material decomposition, especially materials with K-edges in the diagnostic energy range [55]. It has been demonstrated that PCCT can accurately measure calcium, gadolinium, and iodine concentrations in phantoms [56, 57].

PCCT systems are currently under preclinical testing, mostly using phantoms, animal models, *ex vivo* tissue or cadavers. Some authors speculate that due to their improved spectral separation capacity, PCCT could improve the selective recognition and removal of iodine from contrast-enhanced CT images, obtaining more realistic VNC images [53, 58]. Currently, however, density measurements obtained with the first clinical PCCT have a limited diagnostic value. In one study, the liver parenchyma was found to differ by approximately 11 HU between VNC and true non-contrast images [59]. However, the accuracy of PCCT very likely will improve in the coming years. One research established that PCCT could be used to reconstruct phantom and patient VNC images of the liver with accurate attenuation value and without the effects of dose, base material’s attenuation, and liver iodine content [60]. Additionally, a recent research by Niehoff et al. [35] showed that using the spectral datasets obtained from the first clinical PCCT scanner good VNC images could be reconstructed for hepatic steatosis assessment, and all indices showed high sensitivity and specificity even after changing the cut-off values. Despite being the latest technology for CT imaging, PCCT can benefit from further technical advancement to improve its capability to detect and quantify hepatic steatosis.

### Staging of liver fibrosis

**Fig. 3:**
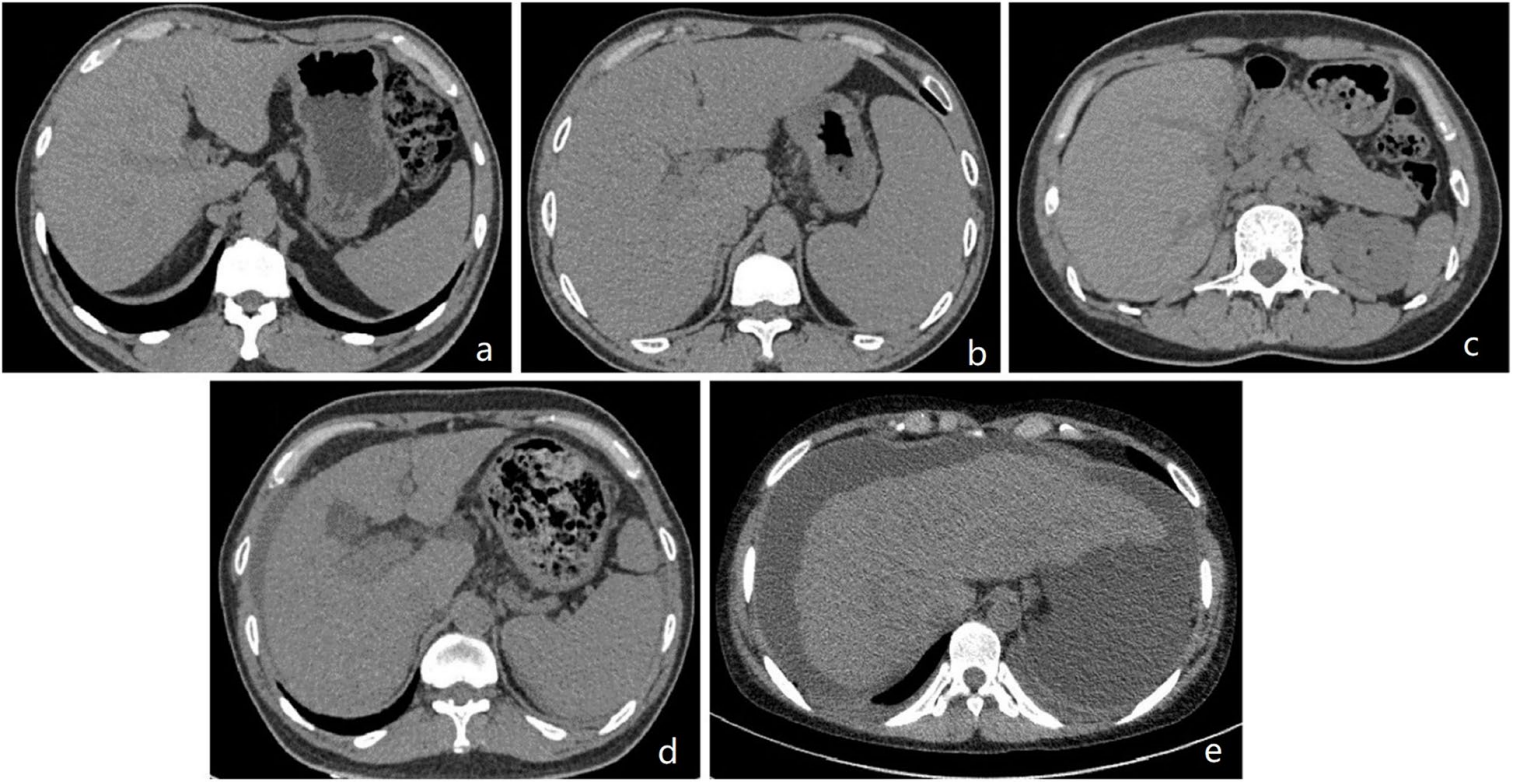
Computed tomography findings of liver fibrosis at each stage. **a** Fibrosis grade 0 (F0): normal liver. **b** Fibrosis grade 1 (F1): no significant change in liver volume and increased volume of spleen. **c** Fibrosis grade 2 (F2): the liver volume is slightly reduced. **d** Fibrosis grade 3 (F3): portal vein thickening, spleen enlargement, and minimal ascites are visible. **e** Fibrosis grade 4 (F4): liver with an irregular shape and ascites are visible

As the degree of hepatic fibrosis is strongly associated with both carcinogenesis and prognosis, a precise assessment is essential for determining its clinical course and prognosis of the patient. For patients with MAFLD, non-invasive diagnosis and staging of liver fibrosis is crucial for assessing disease progression. Techniques such as elastography measure the velocity of the ‘sheer wave’ or tissue displacement due to liver fibrosis to quantify how the organ “stiffers” based on ultrasonic or physical impulse. Ultrasound-based modalities, including vibration-controlled transient elastography, two-dimensional shear wave elastograghy, point shear wave elastography, and magnetic resonance elastography, are advanced elastography technologies for evaluating liver fibrosis. However, increasingly, CT biomarkers are being used to detect and stage hepatic fibrosis (Fig. 3). Current CT methods for detecting liver fibrosis on abdominal CT rely on morphology-based score, contrast-enhanced imaging biomarkers, and post-processing methods. We summarized the CT-based technologies for estimation of hepatic fibrosis (Table 3) and representative studies (Table 4).

**Table 3:** CT methods assessing hepatic fibrosis

Approach	Acronym	Description
Morphology-based score	CRL-R	Caudate-right-lobe ratio = caudate lobe diameter/right lobe diameter
	LIMV-FS	Liver imaging morphology and vein diameter fibrosis score = liver vein diameter/caudate-right-lobe ratio
	LIMA-FS	Liver imaging morphology and attenuation fibrosis score = caudate-right-lobe ratio × liver vein to cava attenuation
	LIMVA-FS	Liver imaging morphology, vein diameter and attenuation fibrosis score = liver vein diameter/caudate-right-lobe ratio × liver vein to cava attenuation
Contrast-enhanced biomarkers	NIC	Normalized iodine concentration = iodine concentration _liver_/ iodine concentration _aorta_ The ICratio was defined as IC_AP_/ IC_PVP_, where IC_AP_ and IC_PVP_ denoted iodine concentrations during AP and PVP, respectively
	ECV	Hepatic extracellular volume—Hounsfield units (%) = △Hounsfield units _liver_ × (100–1-hematocrit (%))/△Hounsfield units _aorta_ △HU_live_ indicates the difference in HUs between the precontrast and equilibrium phase
Postprocessing methods	LSN	A semiautomated postprocessing software
	LSVR	A dedicated computed tomography software tool
	TA	A commercially available texture analysis research software platform
	DLS	The steps for deep learning system: Input CT images $\to$ Liver segmentation algorithm $\to$ Segmented liver images $\to$ Liver fibrosis staging algorithm $\to$ Output
Radiomics		The steps for radiomics: Importing CT images $\to$ ROI segmentation $\to$ Featre extraction $\to$ Feature selection $\to$ Clinical application and analysis

*AP* Arterial phase, *CRL-R* Caudate-right-lobe ratio, *CLD* Caudate lobe diameter, *TA* Texture analysis, *DLS* Deep learning system, *ECV* Hepatic extracellular volume, *HU* Hounsfield units, *Hct* 1-hematocrit, *IC* Iodine concentration, *LSN* Liver surface nodularity, *LSVR* Regional changes in hepatic volume, *LIMV-FS* Liver imaging morphology and vein diameter fibrosis score, *LVD* Liver vein diameter, *LIMA-FS* Liver imaging morphology and attenuation fibrosis score, *LVCA* Liver vein to cava attenuation, *LIMVA-FS* Liver imaging morphology, vein diameter, and attenuation fibrosis score, *NIC* Normalized iodine concentration, *PVP* Portal venous phase, *RLD* Right lobe diameter, *ROI* Circular regions of interest

**Table 4:** Summary of CT studies assessing of hepatic fibrosis

First author [reference]	Number of patients	Methods	Reference standard	AUROC or positive and negative predictive value	Sensitivity	Specificity
Awaya [61]	236	Morphology-based score: CR–L	Pathologically proved cirrhosis	0.797	71.7%	77.4%
Huber [62]	148	Morphology-based score: LIMV-FS	Histologically prove	AUC for cirrhosis is 0.64 AUC for cirrhosis is 0.82	kd/crl-r score ≤ 19.6 for cirrhosis: 88% ld/crl-r score ≤ 23.9 for fibrosis: 83%	kd/crl-r score ≤ 19.688% for cirrhosis: 82% ld/crl-r score ≤ 23.9 for fibrosis: 76%
Obmann [63]	148	Morphology-based score: LIMVA-FS	MR elastography	Any degree of liver fibrosis: 0.84 Significant liver fibrosis: 0.97	Any degree of liver fibrosis: 67% Significant liver fibrosis: 95%	Any degree of liver fibrosis: 88% Significant liver fibrosis: 85%
Lv [64]	81	Contrast-enhanced CT-based imaging biomarkers: NIC	Liver biopsy	ROC of NIC during the PVP: (0.84) and IC_ratio_ (0.65)	NIC: 95% IC_ratio_: 79% Combination of these two parameters: 77%	NIC: 61% IC_ratio_: 49% Combination of these two parameters: 87%
Sofue [65]	47	NIC	Liver biopsy	Each liver fibrosis score (> / = F1–F4): 0.795 to 0855	F0 *versus* F1–4: 75% F0–1 *versus* F2–4: 56.6% F0–2 *versus* F3–4: 57.2% F0–3 *versus* F4:60.8%	F0 *versus* F1–4: 81.4% F0-1 *versus* F2–4: 79.5% F0-2 *versus* F3–4: 81.9% F0-3 *versus* F4: 85.5%
Marri [66]	107	NIC of the right lobe of the liver (RNIC)	Liver biopsy	Metavir fibrosis stage (ranging from F0 to F4): 0.86 to 0.96	F1–F4 fibrosis: 83–93%	F1-F4 fibrosis: 81–87%
Yoon [67]	141	fECV	Liver biopsy	Normal or F0–F1 from F2–F4: 0.832 F4: 0.739	Normal or F0–F1 from F2–F4: 87.5% F4: 73.3%	Normal or F0–F1 from F2–F4: 71.0% F4: 62.7%
Shinagawa [68]	41	ECV-newSub	Liver biopsy	liver fibrosis grades: 0.71	Discrimination of advanced stage (F3–4) from early stage (F0–2): 100%	Discrimination of advanced stage (F3–4) from early stage (F0–2): 100%
Ito [69]	52	ECV	Surgically resected or percutaneously biopsied	ECV_I-B IVC_ cut-off value of 26.4%, discrimination of advanced stage (F3–4) from early stage (F0–2: AUC: 0.95 positive predictive value: 93% negative predictive value: 69%	ECV_I-B IVC_ cut-off value of 26.4%, discrimination of advanced stage (F3–4) from early stage (F0–2: 78%	ECV_I-B IVC_ cut-off value of 26.4%, discrimination of advanced stage (F3–4) from early stage (F0–2: 90%
Yoon [70]	177	ECV-iodine	Liver resection or biopsy	Differentiating F0–1 from F2–4: 0.82 Detecting F4: 0.81	Differentiating F0–1 from F2–4: 82.8% Detecting F4: 74.7%	Differentiating F0–1 from F24: 78.6% Detecting F4: 72.3%
Smith [71]	94	LSN scores	Liver biopsy	Differentiating cirrhotic from noncirrhotic livers: 0.910–0.929	Range: 84–88%	Range: 87–92%
Pickhardt [72]	367	LSN scores	Liver biopsy	F2, F3, F4: 0.902, 0.932, and 0.959, respectively	80.2%; 80.0%; 97.9%	80.0%; 84.2%; 84.8%
Furusato [73]	312	LSVR	Liver biopsy	Distinguishing cirrhosis from normal: 0.916	LSVR ≥ 0.26: 95.4% LSVR ≥ 0.28: 94.4% LSVR ≥ 0.30: 88.0% LSVR ≥ 0.35: 81.5% LSVR ≥ 040: 68.5%	LSVR ≥ 0.26: 51.5% LSVR ≥ 0.28: 63.2% LSVR ≥ 0.30: 71.1% LSVR ≥ 0.26: 88.7% LSVR ≥ 0.40: 96.1%
Pickhardt [74]	624	LSVR	Liver biopsy	F3–F4 *versus* F0–F2: 0.880 F3–F4 *versus* F0–F2: 0.854	F3–F4 *versus* F0–F2: 72.2% F3-F4 *versus* F0–F2: 68.3%	F3–F4 *versus* F0–F2: 88.1% F3–F4 *versus* F0–F2: 87.9%
Lubner [75]	289	TA	Liver biopsy	F0 *versus* F1–4: 0.78 For significant fibrosis (> / = F2): 0.71–0.73 For cirrhosis (> / = F4): 0.86 and 0.87	F0 *versus* F1–4: 74% For significant fibrosis (> / = F2): 71% For cirrhosis (> / = F4): 84%	F0 *versus* F1–4: 74% For significant fibrosis (> / = F2): 68% For cirrhosis (> / = F4): 75%
Yasaka [76]	286	DCNN	Biopsy specimen or surgical specimens	fibrosis (> / = F2): 0.74 fibrosis (> / = F3): 0.76 fibrosis (> / = F3): 0.73	fibrosis (> / = F2): 64% fibrosis (> / = F3): 66% fibrosis (> / = F3): 62%	fibrosis (> / = F2): 85% fibrosis (> / = F3): 85% fibrosis (> / = F3): 84%
Yin [77]	252	LFS network	Liver biopsy	F2–F4: 0.92 F3–F4: 0.89 F4: 0.88	F2–F4: 83.0% F3–F4: 79.5% F4:75.1%	F2–F4: 91.7% F3–F4: 88.2% F4: 86.5%
Wang [78]	332	Radiomic	Liver pathologic examination	F2–F4: 0.904 F3-F4: 0.911 F4: 0.844	F2–F4: 92.1% F3–F4: 83.6% F4: 60.7%	F2–F4: 76.7% F3–F4: 89.3% F4: 95.6%
Yin [79]	252	Radiomic	Histologically proven	F2–F4: 0.92 F3–F4: 0.81 F4: 0.85	Average accuracy rates: F2–F4: 88% F3–F4: 82% F4: 86%	

*AUC* Area under the curve, *CT* Computed tomography, *C/RL* Caudate-right lobe ratio, *TA* Texture analysis, *DCNN* Deep convolutional neural network, *ECV-newSub* ECV obtained from new algorithm data, *ECV* Hepatic extracellular volume, *fECV* Hepatic extracellular volume fractions, *ICratio* Iodine concentration ratio, *LIMV-FS* Liver imaging morphology and vein diameter fibrosis score, *LIMVA-FS* Liver imaging morphology, vein diameter and attenuation fibrosis score, *LFS* Liver fibrosis staging, *LSN* Liver surface nodularity, *LSVR* Regional changes in hepatic volume, *NIC* Normalized iodine concentration, *PVP* Portal venous phase, *ROC* Circular regions of interest

### Morphology-based methods

Quantitative metrics for assessing hepatic fibrosis based on abdominal CT scans are reproducible, require no postprocessing of the images, and can distinguish cirrhotic livers from normal livers with high accuracy. They include caudate-right-lobe ratio (CRL-R) [61], the liver imaging morphology and vein diameter fibrosis score (LIMV-FS) [62], liver imaging morphology and attenuation fibrosis score (LIMA-FS), and liver imaging morphology and vein diameter and attenuation fibrosis score (LIMVA-FS) [63]. And those studies showed that those morphology-based assessments of CT indicators have clinical utility in evaluating the in patients with chronic liver disease, even in the pre-cirrhotic stages of liver fibrosis [61–63]. Notably, enhancement of these scores (LIMVA-FS and LIMA-FS) were better that purely morphology-based CRL-R score [63]. In addition, these quantifiable metrics can be calculated retrospectively on axis planes without time-consuming post-processing and those methods may be easily applied to retrospective CT data analysis. Nonetheless, such linear measurements of liver may not capture all complex changes underlying its morphology.

### Contrast-enhanced biomarkers

CT has limited accuracy in quantifying hepatic fibrosis due to insufficient differences in mass attenuation coefficient between fibrous liver tissue and normal liver tissue. However, fibrosis can be indirectly measured using contrast media as a marker [80]. Markers such as normalized iodine concentration (NIC) and hepatic extracellular volume fraction (ECV) can individually estimate the degree of early hepatic fibrosis in animal and clinical studies. Compared with healthy liver, liver cirrhosis absorb different iodine contrast agents differently during the arterial phase and the venous phase.

NIC, computed as the ratio of liver and aorta contrast concentration during the venous phase, is utilized in DECT imaging to diagnose and stage liver cirrhosis [64–66, 81]. Lv et al. [64] analyzed 38 cirrhosis patients and 43 liver-healthy patients, finding that NIC during the venous phase and the iodine concentration ratio obtained from spectral CT can provide a high level of specificity and sensitivity for distinguishing healthy liver from cirrhotic liver, particularly class C cirrhotic liver. Sofue et al. [65] observed a correlation between NIC in the 3-min delayed DECT scans and severity of liver fibrosis (Spearman *r* = 0.65, *p* < 0.001). However, Marri et al. [66] reported a strong correlation between NIC concentrations in 5-min delayed DECT liver scans and histological forms of liver fibrosis. Based on the rationale that fibrotic areas exhibit a gradual contrast material accumulation, CT acquisition with a delay exceeding 3 min was expected to yield higher iodine concentrations in fibrotic livers. Despite the lack of consensus on the optimal minute for delayed NIC acquisition, NIC using DECT imaging provides a noninvasive method for staging liver fibrosis. The clinical application of DECT iodine measurements for liver fibrosis could be valuable in monitoring disease progression and treatment response, potentially reducing the necessity for liver biopsy.

ECV, which reflects the degree of hepatic fibrosis by measuring the enlarged extracellular space due to collagen fiber deposition, can be assessed during the equilibrium phase of contrast-enhanced CT [82]. The ECV of the liver tissue can be determined using contrast-enhanced CT during the equilibrium phase, when the contrast media has diffused from the intravascular to extravascular spaces to reach an equilibrium. At this contrast-enhanced CT's equilibrium phase, the contrast media is considered to be at equal concentration intravascularly and extravascularly. Consequently, the ECV fraction can be estimated with the following formula: (enhancement in the liver)/(enhancement in the aorta) × (1-hematocrit).

Several studies have validated ECV may act as a reliable biomarker of liver fibrosis [67–70, 83, 84]. Yoon et al. [70] even suggested that ECV is a more suitable parameter for assessing liver fibrosis than iodine density and effective atomic number maps, which are calculated solely based on iodine/water concentration without considering hematocrit levels. They also demonstrated that liver ECV estimated on the basis of HU values showed significant differences between fibrosis stages, but its diagnostic accuracy was lower compared with ECV calculated via iodine density. Despite these promising findings, 3 to 10 min or later delayed phase was used to achieve a consistent steady-state equilibrium condition for ECV measurement in the literature. Further studies are needed to determine the optimal delay time for ECV calculated in the equilibrium phase.

In summary, the use of NIC and ECV with DECT imaging provides valuable insights into hepatic fibrosis evaluation, offering noninvasive alternatives for staging liver fibrosis.

### Postprocessing methods for assessing liver fibrosis

Postprocessing methods for assessing hepatic fibrosis based on CT include liver surface nodularity, liver segmental volume ratio, CT texture analysis (TA), deep learning system (DLS), and radiomics. A quantitative tool developed using a dedicated semiautomated CT software for calculating objective scores of liver surface nodularity was validated for staging hepatic fibrosis [71, 72, 85]. The process of determining the volume of the liver has been made easier by advanced visualization software tools that effectively segment the liver. Several studies showed that liver segmental volume ratio and total splenic volume, which measure CT-based hepatosplenic volumetric changes, can be used for non-invasive staging of liver fibrosis [73, 74]. TA determines the level of heterogeneity in a particular region of interest by analyzing the distribution of pixel and voxel-gray levels in an image based on histogram analysis [86]. Several studies have investigated the application of TA for the assessment of hepatic fibrosis on CT and found that TA parameters are feasible and useful biomarkers for assessing hepatic fibrosis [75, 87]. However, further research is needed to study and standardize TA methodology as TA metrics and software platforms differ widely.

**Fig. 4:**
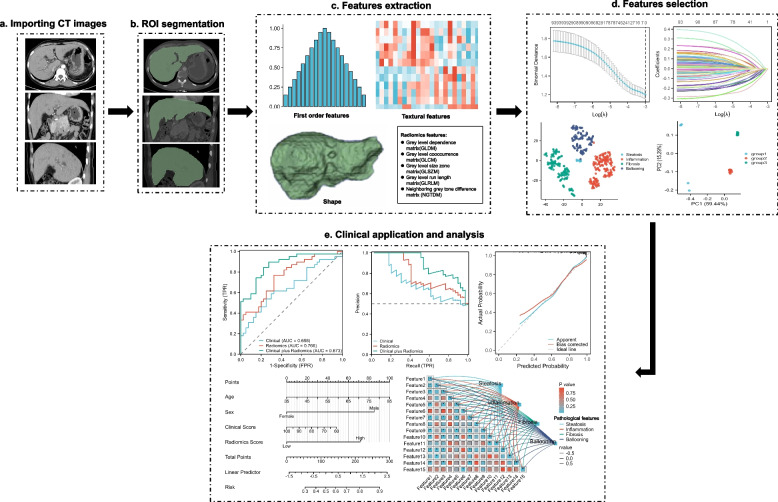
Evaluation of hepatic fibrosis based on radiomics. **a** Importing CT images. **b** The ROI was manually delineated on CT images of the entire liver. **c** First-order statistics, textural features, wavelet or Laplacian of Gaussian transforms, and shape features were extracted. **d** The feature selection is performed using a least absolute shrinkage, selection operator and cluster analysis, and cluster analysis, etc. **e** Nomogram was used to integrate radiomic and clinical features. The performance of established models was evaluated by receiver operator characteristic curve and precision-recall curve, the correlation between pathological features and radiomic features could be also analyzed, etc. *CT* Computed tomography, *ROI* Region of interest

Recently, deep learning methods, specifically neural network with convolutions, have attracted interest as a tool for recognizing and interpreting images. The use of deep learning methods to stage liver fibrosis has been demonstrated in a few studies [24, 76, 77]. DLS provides a promising method for assessing liver fibrosis using CT scans and liver CT scans, which are widely available. Compared with DLS, radiomics analysis requires less data, and computational power is needed for training, as features are extracted from CT scans using manually designed algorithms instead of the raw image. A typical process of hepatic fibrosis evaluation using radiomics is shown in Fig. 4. Additionally, by analyzing radiomic features, radiomics analysis can identify and extract key symptoms that are most relevant to the model from the images, making CT-based radiomics a valuable diagnostic tool for staging liver fibrosis [78]. Another study revealed that incorporating splenic radiomic features and hepatic radiomic features based on CT can improve radiomics analysis for staging liver fibrosis [79].

Although the multiple CT-based biomarkers have been demonstrated as reliable in evaluating liver fibrosis in various mixed and disease-specific cohorts of patients, these techniques are prone to many confounders, such as patient-related factors, operator expertise, technical variations, sampling errors, presence of other liver pathologies, variability in fibrosis distribution and so on. Ideally, hepatic fibrosis should be assessed using a multi-parametric approach that combines the most promising CT features, especially retrospective data acquisition, low cost, and optimal use of resources.

## Conclusions

In summary, MAFLD affects millions of people worldwide, posing a significant burden on economies and healthcare systems. It has become routine clinical practice to assess hepatic steatosis and fibrosis in patients with MAFLD non-invasively. Various CT parameters can be used to identify and stratify the stage of hepatic steatosis and fibrosis with high accuracy. In addition, these methods are attractive due to not only their relationship with hepatic steatosis and fibrosis but also the ease of accessibility and ubiquity of CT technology in clinical settings. With continued improvements in new scanning technique and post-processing method, CT parameters are expected to become more accurate, precise, reproducible, affordable, and routinely applied to non-invasive assessment of hepatic steatosis and fibrosis in MAFLD.

## Data Availability

Not applicable.

## Abbreviations

CT: Computed tomography
DECT: Dual-energy CT
DL: Deep learning
ECV: Extracellular volume
HU: Hounsfield units
MAFLD: Metabolic dysfunction-associated fatty liver disease
MRI: Magnetic resonance imaging
MRI-PDFF: MRI-derived proton density fat fraction
NIC: Normalized iodine concentration
PCCT: Photon-counting CT
QCT: Quantitative CT
TA: Texture analysis
VNC: Virtual non-contrast
